# Impact of an Accessory for Left Ventricular Assist Devices on Device Flow and Pressure Head In Vitro

**DOI:** 10.3390/bioengineering10040486

**Published:** 2023-04-19

**Authors:** Florian Meissner, Derya Eichelkraut, Marius Schimmel, Sven Maier, Heiko Vestner, Manuela Schoen, Martin Czerny, Wolfgang Bothe

**Affiliations:** Department of Cardiovascular Surgery, Medical Center–University of Freiburg, Faculty of Medicine, University of Freiburg, Hugstetter Str. 55, 79106 Freiburg, Germany; florian.meissner@uniklinik-freiburg.de (F.M.); martin.czerny@uniklinik-freiburg.de (M.C.)

**Keywords:** left ventricular assist device, heart failure, mock circulatory loop, in vitro

## Abstract

A novel accessory directing the blood from the outflow of a left ventricular assist device (LVAD) back through the left ventricular apex and across the aortic valve allows LVAD implantation via the left ventricular apex solely but may affect the LVAD performance. We quantified the effect of the accessory on LVAD flow and pressure head in vitro. In a mock circulatory loop, a centrifugal-flow LVAD (HeartMate 3, Abbott, Abbott Park, IL, USA) with (Accessory) and without the accessory (Control) was compared under physiological conditions using a water/glycerol solution as a blood substitute. The pump was operated at 4000, 5200, and 6400 rpm and 5 different resistance levels. Flow, inlet, and outlet pressure were measured, and pressure head was calculated. Compared to the Control, flow and pressure head in the Accessory group were reduced by an overall average of 0.26 L/min and 9.9 mmHg (all speeds and resistance levels). The highest decline in flow and pressure head occurred at the lowest resistance levels. In conclusion, the accessory leads to a reduction of LVAD flow and pressure head that is enhanced by decreases in resistance. Future developments in the LVAD accessory’s design may reduce these effects and allow unimpaired LVAD performance and minimally invasive device implantation.

## 1. Introduction

Patients with terminal heart failure (HF) often require heart transplantation or mechanical circulatory support (MCS). Left ventricular assist devices (LVAD) support the unloading of the left ventricle (LV), increase cardiac output, and improve end-organ perfusion. They are implanted as bridges to transplant and increasingly as destination therapy. Over the past years, centrifugal-flow pumps have replaced axial-flow pumps. New devices have not only become smaller but safer [1]. Nowadays, the most widely implanted LVAD is the HeartMate3 (HM3, Abbott, Abbott Park, IL, USA). Despite steady development, LVAD implantation is still considered a high-risk procedure [2]. In addition to death, stroke, and bleeding, common LVAD-associated adverse events include cardiac arrhythmia, driveline and blood infections, respiratory failure, and right HF. Although rare, external compression of the outflow graft can cause relevant graft obstruction requiring surgical revision or stent implantation [3].

There are various approaches to increase the safety of LVAD implantations. Particularly, minimally invasive implantation techniques, defined as LVAD surgery without sternotomy, have gained importance [2]. LVADs might be implanted via upper hemisternotomy and left anterolateral thoracotomy. In the case of isolated LVAD implantation, off-pump implantation might be considered [4]. With the aim of both minimizing surgical access and off-pump implantation, we are developing an LVAD accessory. The accessory combines LVAD inflow and outflow and can be attached to the actual device. The outflow graft is positioned through the LV across the aortic valve. Thus, transapical access solely is required for LVAD implantation. Recently, we have demonstrated the successful off-pump implantation of a first prototype via left thoracotomy in five pigs [5]. The accessory tested in vivo revealed several rheological limitations. Among others, it had redirections with rather small radii favoring flow turbulences. Furthermore, it was characterized by varying inflow and outflow diameters. The alterations of the cross-sectional area resulted in flow acceleration in more narrow sections and flow deceleration in wider ones. Supported by computational fluid dynamics, we have improved the design of the LVAD accessory continuously. The new prototype is characterized by a flow-optimized design and is made of titanium. The prototype is 3D printed and can be adapted individually to the LV dimensions of the in vivo model. Prospectively, it might be designed individually for the patient undergoing LVAD implantation. In addition to computational fluid dynamics and ahead of in vivo testing, we aimed to assess the performance of the new LVAD accessory with respect to flow, pressure head, and pump power in vitro.

Mock circulatory loops (MCL) are frequently used to assess the performance of MCS devices in vitro. They consist of hydraulic and mechanical components representing defined cardiovascular structures such as the heart and aorta. A broad variety of physiological and pathological conditions (e.g., HF) can be simulated by specific parameters, including systemic resistance and vascular compliance [6]. Although in vitro testing in MCLs is characterized by a high level of standardization and reproducibility, their evidence is limited by the simplification of complex anatomical structures and physiological mechanisms.

In this study, we compared the performance of a continuous-flow HM3 with and without the LVAD accessory in vitro in an MCL using a water/glycerol solution as a blood substitute. Primarily, we aimed to compare both groups in terms of flow, pressure head, and pump power to optimize the accessory’s design further. Secondarily, we aimed to determine the accuracy and reliability of HM3 flow estimation derived from fixed speed, power, and patient’s hematocrit (HCT) as indicators for fluid viscosity [7]. Since the exact algorithm has not been publicly reported by the manufacturer, we aimed to compare measured and estimated flow with and without LVAD accessory in an MCL using a water/glycerol solution instead of human blood.

## 2. Materials and Methods

### 2.1. Mock Circulatory Loop

**Figure 1:**
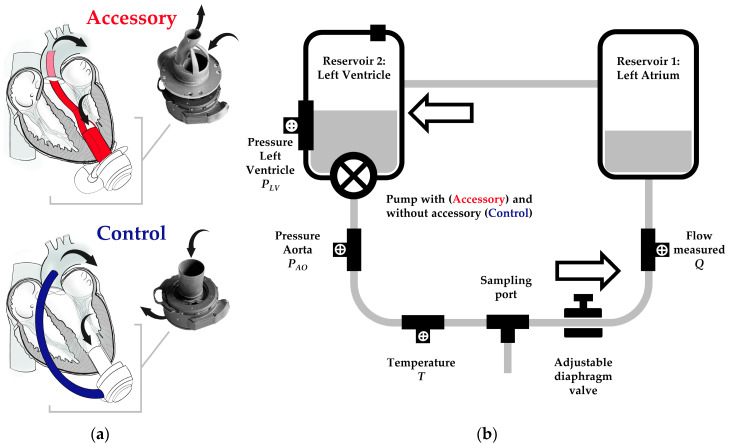
(**a**) Centrifugal-flow left ventricular assist device (LVAD) with and without accessory and (**b**) mock circulatory loop (MCL) schematic. (**a**) The accessory combines the LVAD’s inflow and outflow. Blood from the left ventricle enters the LVAD through the accessory’s inflow (lower arrow). The LVAD ejects the blood towards the accessory’s outflow, which is attached to an outflow graft. The latter is positioned across the aortic valve in the ascending aorta, where the blood leaves the outflow graft (upper arrow). In case of Control and conventional LVADs, the blood is directed from the pump outlet along the anterior outer surface of the heart towards the ascending aorta. (**b**) The MCL consists of two reservoirs representing left atrium and ventricle (Reservoir 1 and 2) and an adjustable diaphragm valve connected by rigid PVC tubing. The LVAD is positioned with or without accessory in Reservoir 2. The inlet pressure is measured in Reservoir 2 ahead of the pump. The outlet pressure is measured behind the LVAD outflow graft. The flow is measured between diaphragm valve and Reservoir 1. In the schematic drawing, the flow direction is counterclockwise.

For in vitro assessment of flow and pressure head of an LVAD with and without accessory (Figure 1a), a simplified MCL was built (Figure 1b). The MCL consisted of two acrylic reservoirs, representing left atrium (LA) and LV. The latter one comprised a site to attach the centrifugal-flow pump with or without LVAD accessory. To control systemic resistance, a diaphragm/membrane valve (GF Piping Systems, Schaffhausen, Switzerland) was used. The reservoirs were connected by rigid 1/2 × 3/32 inch medical-grade polyvinylchloride (PVC) tubing (GF Piping Systems, Switzerland). The pressure was measured continuously in the reservoir representing the LV (referred to as inlet pressure) and behind the centrifugal-flow LVAD (referred to as outlet pressure) with or without LVAD accessory by pressure transducers (RS Pro Pressure Switch G1/4, RS Pro, Frankfurt, Germany). Pressure head was calculated as difference between outlet and inlet pressure. The temperature was measured continuously between LV and diaphragm valve by a temperature sensor (NTC 48 mm, RS Pro, Frankfurt, Germany). The flow was measured between diaphragm valve and LA with a float-type flow meter (M335, SAFI, Taulignan, France). A multi-channel analog-to-digital converter (RedLab 2416-4-AO, Meilhaus Electronic GmbH, Alling, Germany) with proprietary data capture software (DAQami, Meilhaus Electronic GmbH, Alling, Germany) was used for data acquisition at a sampling frequency of 50 Hz. The MCL was filled with 2.5 L of a 60/40 water/glycerol solution (density 1.113 g/cm^2^, approximated dynamic viscosity of 0.0043 Ns/m^2^ at 23 °C) corresponding to an HCT of about 50% [8].

The HM3 was controlled and set using a proprietary system monitor and controller attached to the pump. The HCT value was set at two different levels. An HCT of 50% was set according to the viscosity of the 60/40 water/glycerol solution (constant water/glycerol ratio throughout all experiments). Moreover, an HCT of 20% was set to evaluate the accuracy of the HM3 flow estimation algorithm when applying a wrong or significantly deviating HCT value in case of HM3 the minimum of 20%. Pump speed, power, flow estimate, and pulsatility index (PI) were recorded manually from the system monitor (clinical screen). As shown schematically in Figure 1a, the LVAD accessory was attached to the HM3 inflow. The HM3 outflow was connected to the LVAD accessory by PVC tubing. The diameter of the accessory’s main flow channel in the proximal and distal sections was 12 mm and divided into 2 sub-channels in the middle section with constant cross-sectional area. Different from in vivo testing and scheme drawing, the LVAD accessory was not continued by a 12 mm outflow graft but a PVC tubing of the same diameter.

### 2.2. Experimental Protocol

The HM3 was operated with LVAD accessory (Accessory group) and without (Control group) at 3 rotational speeds (4000, 5200 and 6400 rpm) and 5 resistance levels, which were controlled by an adjustable diaphragm valve. The resistance levels were classified as very low, low, moderate, high, and very high corresponding to a reduction in cross-sectional area within the diaphragm valve by about 15, 35, 55, 65, and 75%. Repeating data acquisition at the lower and upper limits of rotational speed and resistance as well as 1 further for control, 20 experimental runs (3 speeds × 5 resistances + 4 repetition upper/lower limits + 1 repetition median speed/resistance) were acquired per group. Data acquisition lasted for 5 min per run. It was performed in random order to reduce the impact of external factors such as room temperature. For cross-validation, two different HM3 pumps were operated with and without LVAD accessory. In total, 4 series with overall 80 experimental runs (2 groups × 2 cross-validation × 20 per series) were conducted. All experiments were performed at room temperature. The temperature of the water/glycerol solution was not additionally modified.

### 2.3. Statistical Analysis

For each experimental run, flow and HM3 flow estimates as well as LA and LV pressure (inlet and outlet pressure) were reported as means with standard deviations. Between both groups, all parameters were compared by mean difference (MD). Linear regression was performed to compare the flow, pressure head, pump power, and PI between both groups with respect to curve intercepts and slopes. A significance level of 5% and a confidential interval of 95% were applied. All calculations were performed with *R* (R Development Core Team, Vienna, Austria).

## 3. Results

### 3.1. Flow and Pressure Head at Low, Moderate and High Pump Speed

**Figure 2:**
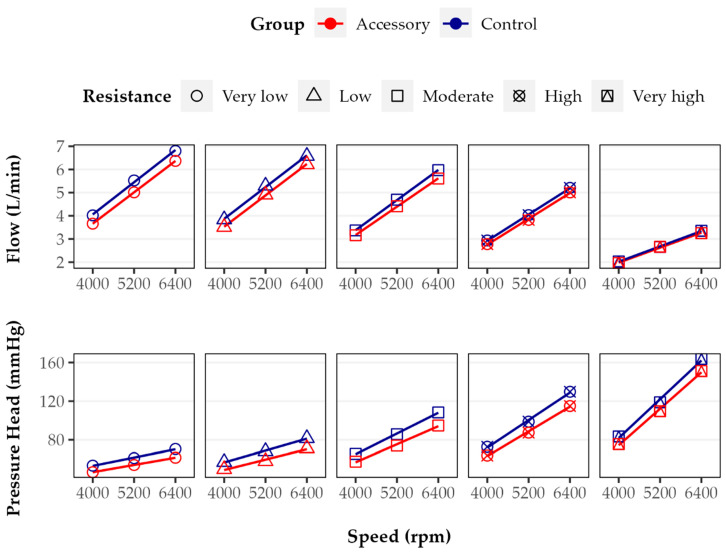
Flow and pressure head of Accessory and Control groups at five different resistance levels (points) with linear regression curves (lines). In both groups, flow and pressure head increased directly proportional to pump speed. Flow and pressure head were lower in the Accessory group than in Control group. While flow decreased with increasing resistance level, pressure head increased.

Flow and pressure head (Figure 2) increased directly proportional to increasing pump speed at all resistance levels. The combination of HM3 with the accessory resulted in a reduced flow and pressure head at almost all tested conditions compared to the Control. With respect to flow, the MD between the Control and Accessory groups was highest at 5200 rpm and very low resistance (MD = 0.51 L/min), which corresponds to a relative difference of 9.3%. With respect to the pressure head, the MD was highest at 6400 rpm and had moderate resistance (MD = 14.7 mmHg) and was lowest at 4000 rpm and had very low resistance (MD = 6.8 mmHg). These values correspond to relative differences of 11.4% and 12.6%, respectively. Overall, when comparing the regression lines of flow and pressure head in terms of curve intercepts and slopes at each resistance level between both groups, we found no significant differences (all *p* > 0.05).

### 3.2. Pressure Head versus Flow (HQ) Curves

**Figure 3:**
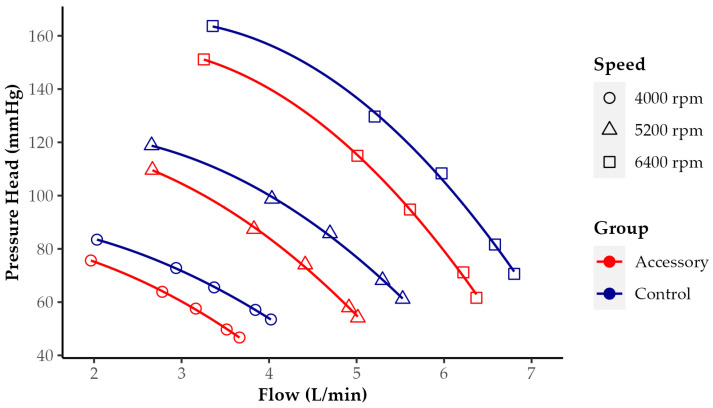
Pressure head versus flow (HQ) curves of HeartMate 3 with and without LVAD accessory at 3 rotational speeds (4000, 5200, and 6400 rpm). Each point in the diagram represents flow and pressure head at one resistance level (at same speed highest flow at lowest resistance). A change in pressure head in the Control group, for example, at 5200 rpm from 100 to 60 mmHg, led to nearly the same change in measured flow as in the Accessory group.

Figure 3 shows the HQ curves of HM3 with and without LVAD accessory at three rotational speeds. In both groups, the HQ relationship is curvilinear with a more negative slope at higher flows. The curves of both groups run parallel to each other. Comparing the Control and Accessory groups at all rotational speeds tested, flow is increased as the pressure head declines, but flow increases higher without an accessory.

### 3.3. Flow and Outlet Pressure Wave Form

**Figure 4:**
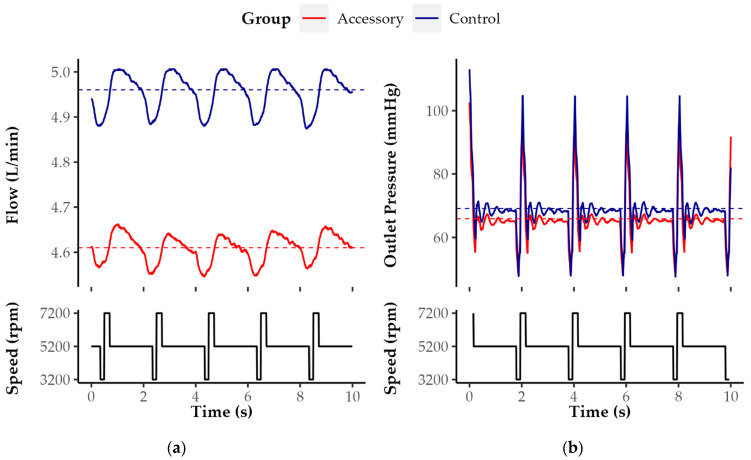
Exemplary (**a**) flow and (**b**) outlet pressure waveform of Control and Accessory groups at 4000 rpm and very low resistance level under artificial pulsatility. The flow and resistance levels were exemplarily selected for illustration according to the greatest relative difference in terms of flow between both groups under these particular conditions.

Flow (Figure 4a) and outlet pressure waveform (Figure 4b) were comparable in both groups with respect to symmetry and frequency. Apparently, mean flow and mean outlet pressure was higher without the LVAD accessory. Furthermore, amplitudes of flow and outlet pressure were slightly decreased in the Accessory group. With and without the accessory, the characteristic periodic curves are the result of artificial pulsatility featured by HM3. At the beginning of each cycle, the pump increases its rotational speed by 4000 rpm for 0.2 s before decreasing it by 2000 rpm. Before the end of each cycle, there is a second decrease by 2000 rpm for 0.15 s. The speed variation was evident in both parameters and groups.

### 3.4. Pump Power and Pulsatility Index (PI)

**Figure 5:**
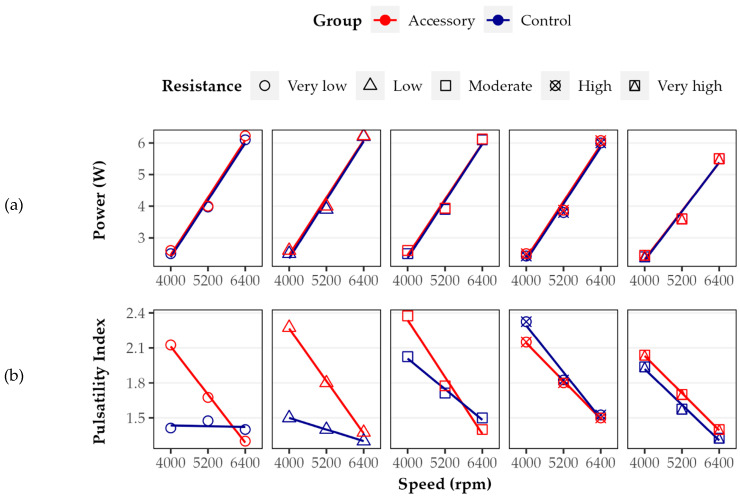
(**a**) Pump power and (**b**) pulsatility index of Accessory and Control groups at five different resistance levels (points) with linear regression curves (lines). While power increased directly proportional to pump speed, pulsatility index decreased. Power and overall pulsatility index were higher in the Accessory group than in Control.

As shown in Figure 5a, in both groups, HM3 power increased directly proportional to pump speed. There were rather small differences between different resistance levels. The greatest difference between the Control and Accessory groups was found at 4000 rpm at a very low to moderate resistance level. Under these conditions, using the LVAD accessory leads to an increase in energy consumption by 4.0%. However, there was no difference at 5200 and a very high resistance level (*p* > 0.05).

As shown in Figure 5b, the PI decreased with increasing pump speed. In the Control group, there was one exception of very low resistance. At 4000 rpm and very low as well as low resistance, the PI was increased in the Accessory group by about 50% compared to the Control group. There were only slight differences between both groups at higher pump speeds and moderate to very high resistance levels. Overall, when comparing the regression lines of power and PI in terms of curve intercepts and slopes at each resistance level between both groups, we found only significant differences in the case of PI at very low and low resistance levels (both *p* < 0.05).

### 3.5. Accuracy of HM3 Flow Estimation

**Figure 6:**
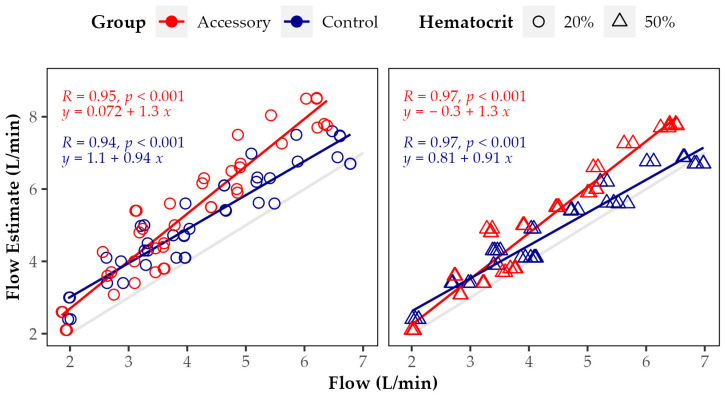
Correlation and linear regression between measured flow and HM3 flow estimates at 20 and 50% preset hematocrit in Accessory and Control groups throughout all rotational speeds and resistance levels. The gray line represents the reference line with measured and estimated flow being equal. Points and regression lines above this reference line indicate flow overestimation by the algorithm applied to estimate the pump flow.

Figure 6 shows the correlation and linear regression between measured flow and HM3 flow estimate at 20 and 50% preset HCT value in both groups. There was a very strong and highly significant correlation between measured and estimated flow at both preset HCT values with and without LVAD accessory (R > 0.9, *p* < 0.05). However, in all cases, the LVAD flow was overestimated. The actually measured flow was always less than the flow estimate. At 50% HCT, the flow estimate was more accurate than at 20% in both groups (R_Accessory_ = 0.95 vs. 0.97, R_Control_ = 0.94 vs. 0.97). In the Control group, both regression curves approach the reference line (measured and estimated flow being equal) with the increasing flow (both slopes < 1). This indicates that the match of measured and estimated flow increases up to the intersection of regression and reference curve at high pump flow. Whereas in the Accessory group, both regression curves move away from the reference with the increasing flow (both slopes > 1), indicating the opposite.

## 4. Discussion

In general, LVAD flow is dependent on rotational speed and inversely dependent on pressure head or differential pressure between the pump inlet and outlet, in clinical use determined by left ventricular and aortic pressure [6]. This study showed that operating HM3 with LVAD accessory reduced flow and pressure head up to 9.3% and 15.0%, respectively.

These findings lead to the question of potential causes, including the accessory’s design. Among others, we identified three main limitations of the current accessory design. First, the accessory reveals two almost rectangular redirections with small radii just behind its inlet. In the case of design optimization, the redirections should be replaced, and the radii, if possible, be increased. Second, in the middle section of the accessory (not shown in detail in Figure 1), the main flow channel is divided into two parallel subchannels. Although the cross-sectional area remains constant, the channel surface area increases, favoring friction loss. Third, in the middle section of the outflow channel, the cross-sectional shape changes from round to oval, which presumably generates turbulences at channel separation and confluence, contributing to flow reduction. This is because consideration should be given to foregoing flow channel separation and changing the cross-sectional shape. The proposed design optimizations may result in a partial loss of the compact and, thus, space-saving design. To achieve an optimal balance between pump performance, characterized by flow and pressure head, and design constraints restricting the size of the implanted device (in particular, due to the limited space between apex and rib cage), the findings from computational fluid dynamics, further MCL tests, and ultimately animal testing must be considered together. A design that has been optimized solely based on in vitro tests does not necessarily correspond to the best design for implantation in vivo.

For in vivo testing, it is additionally important to consider different outflow lengths and graft materials in the Accessory and Control groups. While the length of the accessory’s transventrciular outflow graft depends on the distance between the LV apex and mid-ascending aorta, the length of extracardiac outflow grafts is determined by the anterior shape of the heart, which makes conventional outflow grafts significantly longer (according to our own but not yet published data). For this in vitro study, we decided to apply outflow grafts of equal lengths in both groups. Overall, we hypothesize that the influence of graft length is less relevant than the influence of graft geometry and changes in the cross-sectional area. With respect to the graft material, we apply different materials for MCL and animal testing. In the case of animal testing, we use stent grafts made of well-proven polyester and nitinol materials. These materials have unique mechanical properties but are only limited and suitable for MCL experiments, in particular, due to leakage. This is because we prefer to use PVC tubing with a suitable diameter as an outflow graft in the case of MCL testing. We assume that the influence of different materials is rather small in the case of large-diameter grafts (greater than 8 mm in diameter). Prospectively, we intend to investigate both the effect of different graft lengths as well as the effect of different materials.

Beyond the implications for the accessory’s design, including the outflow graft, it has to be noted that the application of an LVAD accessory, which can be seen as an add-on to the actual LVAD, requires operating the LVAD at a higher rotational speed in order to achieve the same flow and pressure head as the application without an LVAD accessory. Although the differences appear rather small, it cannot be negated that higher pump speeds might contribute to adverse events, for example, increased degradation of hemostatic blood proteins such as von Willebrand factor (vWF). To our knowledge, there has been no study assessing the relationship between HM3 pump speed and vWF degradation in an MCL. However, Bartoli and colleagues investigated the centrifugal-flow EVAHEART pump (EVAHEART, Bellaire, TX) in vitro and found significantly fewer vWF fragments at lower pump speeds as a result of less shear stress [9]. On the other hand, studies on axial-flow HeartMate II (Abbott, Abbott Park, IL, USA), the pump generation before centrifugal-flow HM3, show that reduced pump speed does not significantly decrease vWF degradation [10]. In the absence of data for HM3, however, we hypothesize that blood trauma increases when the use of an LVAD accessory requires higher pump speeds. To determine the exact relationship between HM3 pump speed and vWF degradation, further investigations are necessary.

Each LVAD pump has its unique pressure head–flow relationship, also referred to as pump or HQ curve. Since for HM3, the HQ curves with and without LVAD accessory are curvilinear and parallel, the same change in flow results in almost the same change in pressure head. From a clinical perspective, the accessory seems to have no relevant impact on the pump’s sensitivity to afterload as well as higher blood pressure [11]. Compared to the reference HQ curves for HM3 reported in intervals of 1000 rpm by the manufacturer [12], there are slight differences to the HQ curves obtained for HM3 without the accessory in this study. Although all curves are curvilinear, the negative slopes of the reference curves are smaller. Therefore, the same increase in pressure head leads to a higher increase in flow [13]. This might be explained by differences in the experimental setup of the MCL including pre- and afterload.

The pump’s power consumption is the product of current and voltage applied to the motor. In the case of HM3, it ranges from 3 to 7 W [11]. During our in vitro testing, the lowest pump power was found at the lowest resistance level and pump speed without the accessory. In both groups, the power at least doubled while increasing the pump speed from 4000 to 6400 rpm but slightly decreased with higher resistance. The relative difference found with and without the accessory was rather small, ranging from 0.0 to 4.0%. Taking into consideration that a fully charged set of batteries powers the system for 10 to 17 h, this duration would be reduced by about 25 to 40 min.

In clinical use, the PI quantifies the flow variation across the cardiac cycle. It is calculated from maximum and minimum flow as well as mean flow (PI = [Maximum − Minimum]/Mean Flow). In the case of HM3, it ranges between 1 and 4 [11]. Our results indicate that in both groups the PI decreases with increasing pump speed. The PI decreases most likely due to the increase in mean flow.

Referring to the accuracy of HM3 flow estimation with and without the accessory, our data show that there is a strong and highly significant correlation between measured and estimated flow. However, overall, the flow estimate is significantly higher than the measured flow, in particular, when applying an HCT value of 20%. The relative overestimation increased with higher resistance, higher pump speed, and with the LVAD accessory. The greatest relative difference between measured and estimated flow was found at 6400 rpm and very high resistance with LVAD accessory and at low HCT. Since the HM3 flow estimation algorithm does not account for the use of an accessory, which according to our results, reduces flow and pressure head, this might explain, in part, the observed increased flow overestimation in the Accessory group. Overall, the reliability of HM3 flow estimation in an MCL without appropriate correction is limited. To our knowledge, so far, the exact algorithm applied for HM3 flow estimation has not been reported. Nevertheless, according to the manufacturer, it is derived from fixed speed, power, and the patient’s HCT or preset viscosity. Furthermore, the flow estimate is reduced by increased blood pressure [7]. As in our MCL, the first in vitro studies evaluating the algorithm’s accuracy were performed with a water/glycerol solution. For flows greater than 3 L/min, the accuracy was about 10%, which is comparable to the results of our study at a very low resistance level throughout all tested pump speeds without the accessory [14]. Analogous to Hasin and colleagues for HeartMate II, Castagna and colleagues found only a poor correlation between HM3 flow estimate and the cardiac output measured by thermodilution (R = 0.46, *p* < 0.0001). The authors proposed to include LVAD power, systolic blood pressure, hemoglobin, and weight in the algorithm applied for flow estimation [15].

Addressing the limitations of this study, the MCL used to determine the impact of the LVAD accessory on flow and pressure head was simplified. For example, the remaining heart function and physiological pulsatility as well as vascular compliance were not considered. Furthermore, the MCL did not account for HF and specific hemodynamic conditions in particular. The diaphragm valve could be adjusted only gradually, which made precise adjustment difficult. Instead of whole blood, a water/glycerol solution was applied as fluid. Lastly, all experiments were conducted at room temperature (about 23 °C) instead of body temperature.

## 5. Conclusions

This study investigated the impact of an LVAD accessory with transaortic outflow graft on HM3 flow and pressure head in vitro. The accessory reduced flow and pressure head of the LVAD. The relative reduction of both was enhanced by decreases in resistance. Future developments in the accessory’s design may further reduce this effect and allow unimpaired LVAD performance and minimally invasive device implantation.

## Data Availability

The data supporting this article’s findings are available from the corresponding author, Wolfgang Bothe [wolfgang.bothe@uniklinik-freiburg.de].

## References

[1] Mehra M.R., Uriel N., Naka Y., Cleveland J.C., Yuzefpolskaya M., Salerno C.T., Walsh M.N., Milano C.A., Patel C.B., Hutchins S.W. (2019). A Fully Magnetically Levitated Left Ventricular Assist Device—Final Report. N. Engl. J. Med..

[2] Ribeiro R.V.P., Lee J., Elbatarny M., Friedrich J.O., Singh S., Yau T., Yanagawa B. (2022). Left Ventricular Assist Device Implantation via Lateral Thoracotomy: A Systematic Review and Meta-Analysis. J. Heart Lung Transplant..

[3] Wert L., Stewart G.C., Mehra M.R., Milwidsky A., Jorde U.P., Goldstein D.J., Selzman C.H., Stehlik J., Alshamdin F.D., Khaliel F.H. (2022). A Multicenter Evaluation of External Outflow Graft Obstruction with a Fully Magnetically Levitated Left Ventricular Assist Device. J. Thorac. Cardiovasc. Surg..

[4] Hanke J.S., Rojas S.V., Avsar M., Haverich A., Schmitto J.D. (2015). Minimally-Invasive LVAD Implantation: State of the Art. Curr. Cardiol. Rev..

[5] Schibilsky D., Scheumann J., Koester P.J., Demir H., Rausch M., Puiu P., Benk C., Maier S., Neudorf S., Diel P. (2022). Development of a Novel Adapter to Enable Less-Invasive Left Ventricular Assist Device Implantation via the Left Ventricular Apex. ASAIO J..

[6] Noor M.R., Ho C.H., Parker K.H., Simon A.R., Banner N.R., Bowles C.T. (2016). Investigation of the Characteristics of HeartWare HVAD and Thoratec HeartMate II Under Steady and Pulsatile Flow Conditions: LVAD and Mock Circulation. Artif. Organs.

[7] Abbott HeartMate 3TM Left Ventricular Assist Device: Pump Parameter Overview. https://www.cardiovascular.abbott/content/dam/bss/divisionalsites/cv/hcp/education-training/heart-failure/documents/hf-heartmate3-lvad-pump-parameters.pdf.

[8] Boës S., Ochsner G., Amacher R., Petrou A., Meboldt M., Schmid Daners M. (2018). Control of the Fluid Viscosity in a Mock Circulation: Viscosity Control for a Mock Circulation. Artif. Organs.

[9] Bartoli C.R., Kang J., Motomura T. (2020). Decreased RPM Reduces von Willebrand Factor Degradation with the EVAHEART LVAS: Implications for Device-specific LVAD Management. J. Card. Surg..

[10] Kang J., Zhang D.M., Restle D.J., Kallel F., Acker M.A., Atluri P., Bartoli C.R. (2016). Reduced Continuous-Flow Left Ventricular Assist Device Speed Does Not Decrease von Willebrand Factor Degradation. J. Thorac. Cardiovasc. Surg..

[11] Tchoukina I., Smallfield M.C., Shah K.B. (2018). Device Management and Flow Optimization on Left Ventricular Assist Device Support. Crit. Care Clin..

[12] Abbott HeartMate 3TM Left Ventricular Assist System: Instructions for Use. https://www.accessdata.fda.gov/cdrh_docs/pdf16/P160054C.pdf.

[13] Heatley G., Sood P., Goldstein D., Uriel N., Cleveland J., Middlebrook D., Mehra M.R. (2016). MOMENTUM 3 Investigators Clinical Trial Design and Rationale of the Multicenter Study of MagLev Technology in Patients Undergoing Mechanical Circulatory Support Therapy With HeartMate 3 (MOMENTUM 3) Investigational Device Exemption Clinical Study Protocol. J. Heart Lung Transplant. Off. Publ. Int. Soc. Heart Transplant..

[14] Hahn J., Loree H., Cernes D., Poirier V., Richardson S., Fleischli A., Gempp T., Schoeb R., Litwak K., Kameneva M. (2002). HeartMate III Dvelopment of a Sensorless Flow Estimation Algorithm. ASAIO J..

[15] Castagna F., Mondellini G.M., Braghieri L., Kim A., Takeda K., Naka Y., Sayer G.T., Uriel N., Yuzefpolskaya M., Colombo P.C. (2021). Estimation of Total Cardiac Output Using Non-Invasive Parameters in HeartMate 3 Patients. J. Heart Lung Transplant..

